# Lys63/Met1-hybrid ubiquitin chains are commonly formed during the activation of innate immune signalling

**DOI:** 10.1016/j.bbrc.2016.04.141

**Published:** 2016-06-03

**Authors:** Christoph H. Emmerich, Siddharth Bakshi, Ian R. Kelsall, Juanma Ortiz-Guerrero, Natalia Shpiro, Philip Cohen

**Affiliations:** MRC Protein Phosphorylation and Ubiquitylation Unit, School of Life Sciences, University of Dundee, Dundee DD1 5EH, Scotland, UK

**Keywords:** Ubiquitin, Innate immunity, TNF, TLR3, NOD1, LUBAC, AMSH-LP, AMSH-like protein, λ-PPase, bacteriophage λ protein phosphatase, BMDM, bone marrow-derived macrophages, JNK, c-Jun N-terminal kinase, cIAP, cellular Inhibitor of Apoptosis, DUB, deubiquitylase, dsRNA, double-stranded RNA, GST, glutathione-S-transferase, HRP, horseradish peroxidase, IKK, IκB kinase, IL-1, interleukin-1, IL-1R, IL-1 receptor, IRAK, IL-1R-Associated Kinase, LUBAC, Linear UBiquitin Assembly Complex, K63-Ub, Lys63-linked ubiquitin, M-CSF, Macrophage Colony Stimulating Factor, M1-Ub, Met1-linked ubiquitin, MyD88, Myeloid Differentiation primary response gene 88, NEMO, NF-κB Essential Modifier, NOD, Nucleotide Oligomerisation Domain, PRRs, Pathogen Recognition Receptors, PNGase F, Protein Asparaginyl Glycosidase F, RIP, Receptor-Interacting Protein, TAK1, TGFβ-activated kinase 1, TAB, TAK1-binding protein, TRAF, TNF-Receptor-Associated Factor, TLR, Toll-Like Receptor, TRIF, TIR-domain-containing adapter-inducing interferon-β, TNFα, Tumour Necrosis Factor α, TNFR1, TNFα Receptor 1, TRADD, TNF-receptor associated death domain protein, Ub, Ubiquitin, USP, Ub-specific protease

## Abstract

We have reported previously that activation of the MyD88-signaling network rapidly induces the formation of hybrid ubiquitin chains containing both Lys63-linked and Met1-linked ubiquitin (Ub) oligomers, some of which are attached covalently to Interleukin Receptor Associated kinase 1. Here we show that Lys63/Met1-Ub hybrids are also formed rapidly when the TNFR1/TRADD, TLR3/TRIF- and NOD1/RIP2-signaling networks are activated, some of which are attached covalently to Receptor-Interacting Protein 1 (TNFR1 pathway) or Receptor-Interacting Protein 2 (NOD1 pathway). These observations suggest that the formation of Lys63/Met1-Ub hybrids are of general significance for the regulation of innate immune signaling systems, and their potential roles in vivo are discussed. We also report that TNFα induces the attachment of Met1-linked Ub chains directly to TNF receptor 1, which do not seem to be attached covalently to Lys63-linked or other types of ubiquitin chain.

## Highlights

• Ubiquitin chains containing both Lys63 and Met1 linkages are commonly formed during innate immune signaling.
• Lys63/Met1-hybrid chains become attached to RIP1 and RIP2 in the TNFR1 and NOD1 signaling networks, respectively.
• Potential advantages of Lys63/Met1-hybrids over separate ubiquitin chains are proposed.
• Met1-linked ubiquitin is attached to TNFR1 without formation of a hybrid ubiquitin chain.

## 1 Introduction

The innate immune system is vital for defense against infection by microbial pathogens, especially in young children [1]. In this system, components of these microbes activate Pathogen Recognition Receptors, such as TLRs and NOD proteins, triggering the production of pro-inflammatory cytokines, such as IL-1 and TNFα, which mount responses to combat the invading microbes.

Most TLRs, as well as the IL-1R, initiate signal transduction by recruiting the adaptor protein MyD88, which is followed by the binding of IRAK4 to MyD88, and then the association of other IRAK family members with IRAK4 to form an oligomeric complex, termed the Myddosome [2], [3]. This leads within minutes to the interaction of IRAKs 1 and 2 with the E3 ubiquitin ligase TRAF6 [4], [5] and to the formation of Lys63-linked ubiquitin and Met1-linked ubiquitin chains (K63-Ub, M1-Ub chains). The M1-Ub chains (also called linear Ub chains) are formed by the E3 ligase LUBAC [6], while K63-Ub chains can be formed by the action of TRAF6 in combination with the E2-conjugating complex Ubc13-Uev1a (also called UBE2N-UBE2V1) [7], [8].

We found that the M1-Ub chains formed upon activation of the MyD88 signaling network are attached covalently to preformed K63-Ub chains, forming ubiquitin chains containing both types of linkage, hereafter called K63/M1-Ub “hybrids” [9]. Some of the K63/M1-Ub hybrids present in extracts prepared from IL-1 receptor-expressing HEK293 cells (IL-1R cells) or human THP1 monocytes were attached to IRAK1, but some were not anchored to any other protein [9]. HOIP, the catalytic subunit of LUBAC, interacts with K63-Ub oligomers specifically, but not with M1-Ub oligomers [9], which may help to explain, at least in part, why K63-Ub oligomers (and not ubiquitin monomers) are the preferred substrate for LUBAC in the MyD88-dependent signaling network.

The formation of K63/M1-Ub hybrids provides a platform for the co-recruitment of two or more proteins that bind specifically to either K63-Ub or M1-Ub oligomers, which include the two “master” protein kinase complexes of the MyD88 signaling network, the TAK1 and canonical IKK complexes. The TAB2 and TAB3 components of TAK1 complexes [10] interact specifically with K63-Ub oligomers [11], [12] while NEMO, a regulatory component of the canonical IKK complex, binds to M1-Ub dimers with far higher affinity than it binds to K63-Ub dimers [13], [14]. We have suggested that the co-recruitment of these kinases to K63/M1-Ub hybrids may increase the efficiency with which TAK1 initiates activation of the IKK complex [15].

Met1-linked ubiquitin chains are also formed when other innate immune signaling networks are activated, such as the TNFα [16] and NOD2 [17] signaling pathways and, similar to the MyD88 pathway, activation of TNFR1 or NOD2 induces the activation of TAK1 and the canonical IKK complex. However, whether the M1-Ub chains formed when other innate immune signaling pathways are activated become attached covalently to K63-Ub and/or other types of ubiquitin linkage, has not yet been investigated. Here we demonstrate the rapid formation of K63/M1-Ub hybrids when TNFR1, TLR3 and NOD1 signaling is activated, indicating that the production of these molecules is a general feature of innate immune signaling pathways.

## 2 Methods

### 2.1 Proteins

Proteins were of human origin and full length, unless stated otherwise and were expressed in *Escherichia coli* and purified by the Protein Production Teams of the MRC Protein Phosphorylation and Ubiquitylation Unit (MRC-PPU) coordinated by James Hastie and Axel Knebel. The proteins were:- λPPase (DU4170), GST-Otulin (DU43487), AMSH-LP[264–436] (DU15780), vOTU (DU45351), GST-OTUD3 (DU21323), His_6_-TRABID[245–697] (DU22468), GST-Cezanne (DU20899), OTUB1 (DU19741) and Rat USP2[271–618] (DU35832). The expression vectors and proteins generated with their assigned [DU] numbers can be ordered from the reagent's section of the MRC-PPU website (https://mrcppureagents.dundee.ac.uk/). Murine TNFα was obtained from Peprotech (#315-01A) and poly(I:C) from InvivoGen (tlrl-pic). Lys63-linked (K63_2-7_) and Met1-linked (M1_2-7_) ubiquitin oligomeric standards were purchased from Boston Biochem. The Halo-NEMO and Halo-TAB2 beads were prepared [9] and the NOD1 agonist KF1B synthesized as described [18], [19]. PNGase F was from New England Biolabs (#P0704S).

### 2.2 Antibodies

An antibody recognizing IKKα phosphorylated at both Ser176 and Ser180 and IKKβ phosphorylated at Ser177 and Ser181 (#2697) and antibodies recognizing IRAK1 (#4504), K63-Ub linkages (#5621) and GAPDH (#2118) were from Cell Signaling Technology. A phospho-specific antibody recognizing JNK1 and JNK2 phosphorylated at Thr183 and Tyr185 (#44682) was obtained from Invitrogen and an anti-RIP2 antibody from Abcam (ab8428). Anti-ubiquitin was from Dako (#Z0458) and anti-RIP1 (#610459) from BD Biosciences. An antibody recognizing M1-Ub chains specifically [20] was generously provided by Vishva Dixit, Genentech, USA. Anti-TNFR1 (sc-8436) was from Santa Cruz. Secondary antibodies coupled to HRP were from Thermo Scientific.

### 2.3 Mice, cell culture, cell stimulation and cell lysis

Heterozygous knock-in mice expressing an E3-ligase inactive mutant of HOIP (HOIP[C879S]) [9] were crossed to TNFR1 knock-out mice. Macrophages were obtained by differentiation of foetal livers from E13.5 embryos or bone marrow obtained from the femur and tibia of mice, as described [4]. Adherent BMDM were re-plated into 12-well tissue culture plates (5 × 10^5^ cells) or 10 cm tissue culture grade plates (5 × 10^6^ cells) using fresh culture medium. After re-plating, liver macrophages or BMDM were stimulated with 10 μg/ml poly(I:C) or RAW macrophages with 25 μM KF1B. Monocytes were purified from human peripheral blood mononuclear cells and differentiated into macrophages as described [21]. The human monocyte cell line THP1 was maintained in RPMI medium supplemented with 5% foetal bovine serum (FBS), 2 mM L-glutamine and antibiotics (100 Units/ml penicillin, 0.1 mg/ml streptomycin) and cultured at 37 °C in an 8% CO_2_ humidified atmosphere. HeLa cells and MEFs were cultured in Dulbecco's modified Eagle's medium (DMEM). THP-1 and HeLa cells were stimulated with 10 ng/ml human TNFα or MEFs with 5 ng/ml mouse IL-1α or 10 ng/ml mTNFα.

The cells were rinsed in ice-cold PBS and, unless stated otherwise, were extracted in ice cold lysis buffer (50 mM Tris/HCl pH 7.5, 1 mM EGTA, 1 mM EDTA, 1% (v/v) Triton X-100, 1 mM sodium ortho-vanadate, 50 mM NaF, 5 mM sodium pyrophosphate, 0.27 M sucrose, 10 mM sodium 2-glycerophosphate, 1 mM phenylmethylsulphonyl fluoride, 1 mM benzamidine, plus 100 mM iodoacetamide to inactivate deubiquitylase activities. Cell lysates were clarified by centrifugation at 14,000 × g for 30 min at 4 °C and the supernatants (cell extracts) were collected and their protein concentrations determined by the Bradford procedure.

### 2.4 Capture of ubiquitin chains or ubiquitylated proteins

To capture ubiquitin chains, ubiquitylated proteins and other proteins with which they interact, cell extracts (2–3 mg protein) were incubated overnight at 4 °C with either Halo-NEMO or Halo-TAB2 beads (20 μl packed volume) as described [9], [22]. The beads were washed three times with 1 ml lysis buffer containing 500 mM NaCl and once with 1 ml 10 mM Tris/HCl pH 8.0 and the captured proteins were released by denaturation in SDS. To analyze ubiquitylation events triggered by the NOD1 agonist KF1B, Halo-NEMO beads were first washed twice, each time for 3 min, with 50 mM Tris/HCl pH 7.5 containing 0.1% (w/v) SDS and then three times with 50 mM Tris/HCl pH 7.5, 500 mM NaCl, 1% (v/v) Triton X100, and once with 50 mM Tris/HCl pH 7.5. The brief wash with 0.1% (v/v) SDS did not interfere with the capture of ubiquitin chains and ubiquitylated proteins from the cell extracts but removed an impurity in the bacterially expressed Halo-NEMO preparation that was recognized by anti-RIP2 and was of similar molecular mass to RIP2.

### 2.5 Treatment with deubiquitylases, phosphatase and PNGase F

Proteins captured by Halo-NEMO beads were washed three times with 1 ml of lysis buffer containing 0.5 M NaCl and once with 1 ml of 50 mM Tris/HCl pH 7.5, 50 mM NaCl, 2 mM DTT. The beads were resuspended in 30 μl of 50 mM HEPES pH 7.5, 100 mM NaCl, 2 mM dithiothreitol (DTT), 1 mM MnCl_2,_ 0.01% (w/v) Brij-35) with λPPase (100 units/reaction) and incubated for 1 h at 37 °C with or without the deubiquitylases (DUBs) USP2 (1.0 μM), AMSH-LP (0.2 μM), vOTU (0.1 μM), Otulin (1.0 μM), OTUB1 (2.0 μM), Cezanne (5.0 μM), OTUD3 (2.0 μM) or TRABID (1.0 μM). To deglycosylate TNFR1, SDS and DTT were added to final concentrations of 0.5% (w/v) and 40 mM, respectively, after DUB treatment and heated for 10 min at 100 °C. After cooling to 21 °C, NP-40 and sodium phosphate buffer pH 7.5 were added to final concentrations of 1% (v/v) and 50 mM, respectively, followed by incubation for 1 h at 37 °C with PNGase F (500 Units per reaction). Incubations were terminated by denaturation in LDS and eluted proteins separated from the beads using Spin-X columns and subjected to SDS-PAGE.

### 2.6 Immunoblotting

This was performed using the ECL system (GE Healthcare).

## 3 Results

### 3.1 TNFα induces the formation of K63/M1-Ub hybrids in human THP1 monocytes

**Fig. 1:**
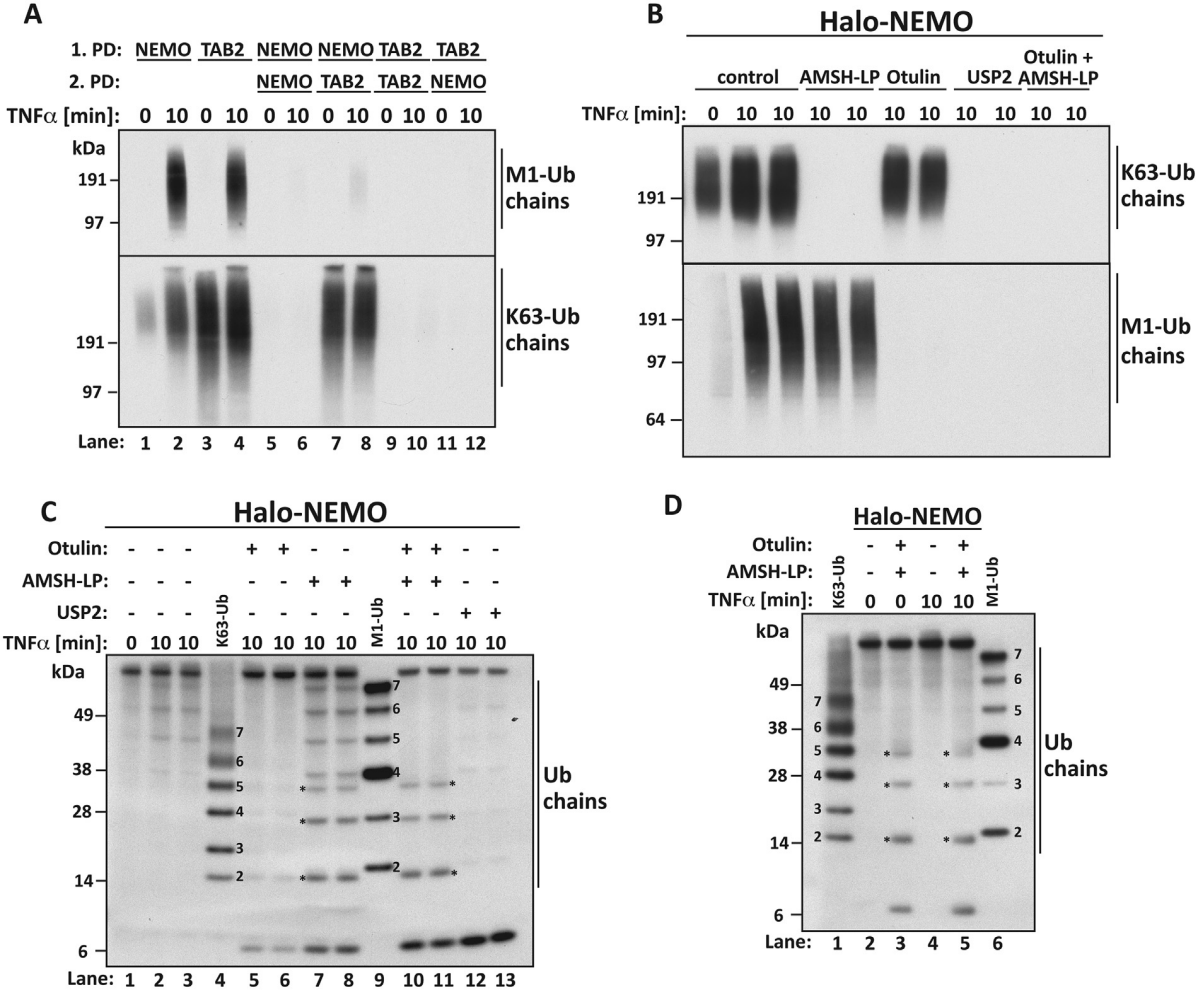
**TNFα-stimulation of human THP1 monocytes induces the formation of K63/M1-Ub hybrids**. (**A**) THP-1 monocytes were stimulated for 10 min with 10 ng/ml hTNFα and ubiquitin chains were captured from 2 mg of cell extract protein with Halo-NEMO or Halo-TAB2 [first pulldown (1.PD)]. The supernatants from the first PD were subjected to a second PD (2.PD) using either Halo-NEMO or Halo-TAB2 as indicated. Captured Ub chains were identified by immunoblotting with antibodies that recognize K63-Ub or M1-Ub chains specifically. (**B**) As in (A) except that, after the Halo-NEMO pull-down, samples were treated for 1 h with phage λPPase (100 units) in the absence (control) or presence of the deubiquitylases AMSH-LP (0.2 μM), Otulin (1.0 μM), AMSH-LP plus Otulin, or USP2 (1.0 μM). The samples were subjected to SDS/PAGE and immunoblotted with antibodies recognizing K63-Ub or M1-Ub chains. (**C, D**) As in B, except that an anti-Ub antibody recognizing M1-Ub and K63-Ub linkages equally well was used for immunoblotting to detect the small Ub oligomers released by treatment with DUBs. In addition, K63-Ub oligomeric markers (5 ng/lane) and M1-Ub oligomeric markers (12 ng/lane) were included in Lanes 4 and 9 in C and lanes 1 and 6 in D to identify the small Ub oligomers formed after treatment with AMSH-LP and/or Otulin.

We stimulated THP1 cells with TNFα and captured the M1-Ub chains and K63-Ub chains formed after 10 min on Halo-NEMO beads or Halo-TAB2 beads (Fig. 1A). The immobilized Halo-NEMO beads capture all the M1-Ub chains present in the cell extracts, as shown by the failure of fresh Halo-NEMO beads to capture any more M1-Ub chains from the supernatant obtained after the first Halo-NEMO pull down (Fig. 1A, top panel compare lanes 2 and 6). The Halo-NEMO beads also captured a small proportion of the K63-Ub chains present in the cell extracts (Fig. 1A, bottom panel lanes 1 and 2), which should include any K63-Ub chains attached covalently to M1-Ub chains as K63/M1-Ub hybrids. In contrast, Halo-TAB2 beads, which bind to K63-Ub chains specifically and do not interact directly with M1-Ub chains [11], [12], captured all the K63-Ub chains present in the cell extracts (Fig. 1A, bottom panel compare lanes 3 and 4 with 9 and 10). Interestingly, the Halo-TAB2 beads also captured all the M1-Ub chains present in the cell extracts (Fig. 1A, compare lanes 4 and 12). This is consistent with the notion that the M1-Ub chains may be attached covalently to K63-Ub chains.

To investigate whether TNFα had induced the formation of K63/M1-Ub hybrids, we studied the formation of the small ubiquitin oligomers that were generated when Halo-NEMO beads were incubated with the AMSH-LP), a DUB that cleaves K63-Ub chains specifically [23] or with Otulin, a DUB that only cleaves M1-Ub chains [24]. These DUBs cleaved all the large K63-Ub chains or M1-Ub chains captured by Halo-NEMO beads (Fig. 1B). Similar to our previous findings in the IL-1 signaling network [9], we found that the complete hydrolysis of K63-Ub chains induced the appearance of small Ub oligomers containing 4–7 ubiquitins, which co-migrated with M1-Ub standard oligomers (Fig. 1C, compare lanes 7–9 with lanes 5 and 6) and could be hydrolysed by Otulin (Fig. 1C, compare Lanes 10 and 11 with lanes 7 and 8). These findings establish that TNFα stimulates the formation of K63/M1-Ub hybrids within minutes in THP1 monocytes.

Incubation of the Halo-NEMO beads with AMSH-LP also generated small ubiquitin oligomers containing 2, 3 and 4 ubiquitins (indicated by stars) but their electrophoretic mobilities differed from either K63-Ub or M1-Ub oligomers of the same chain length (Fig. 1C, lanes 7 and 8). These Ub oligomers were resistant to hydrolysis by both AMSH-LP or Otulin but could be cleaved by USP2 (Fig. 1C, lanes 10–13). These small Ub-oligomers were generated in similar amounts by incubation with AMSH-LP plus Otulin, whether or not the cells had been stimulated with TNFα (Fig. 1D, lanes 3 and 5). They are presumably derived from a hybrid ubiquitin chain comprising K63-Ub and another type of ubiquitin linkage, which is present in the extracts of cells not stimulated with TNFα. Why these hybrid ubiquitin chains are captured by Halo-NEMO is unclear.

The hydrolysis of M1-Ub chains also led consistently to the formation of small Ub oligomers containing 4–6 ubiquitins that co-migrated with K63-Ub standard oligomers during SDS-PAGE and were hydrolysed by AMSH-LP (Fig. 1C compare lanes 4–6 with lanes 10 and 11). However, they were difficult to detect by immunoblotting, presumably due to the paucity of these K63-Ub oligomers.

### 3.2 TNFα-induces the formation of hybrid ubiquitin chains attached to RIP1

TNFα-stimulation induces the recruitment of the adaptor protein TRADD to TNFR1, which is followed by the recruitment of a signaling complex containing TRAF2, the E3 ubiquitin ligases cIAP1) and cIAP2, Receptor-Interacting Protein 1 (RIP1) and LUBAC [25]. The cIAP1 and cIAP2 catalyze the ubiquitylation of RIP1, and the ubiquitin chains attached to RIP1 are thought to bind to TAB2 and TAB3 and induce activation of the TAK1 complex. TAK1 can then activate the canonical IKK complex and MAP kinase cascades [11], [26], [27], [28].

**Fig. 2:**
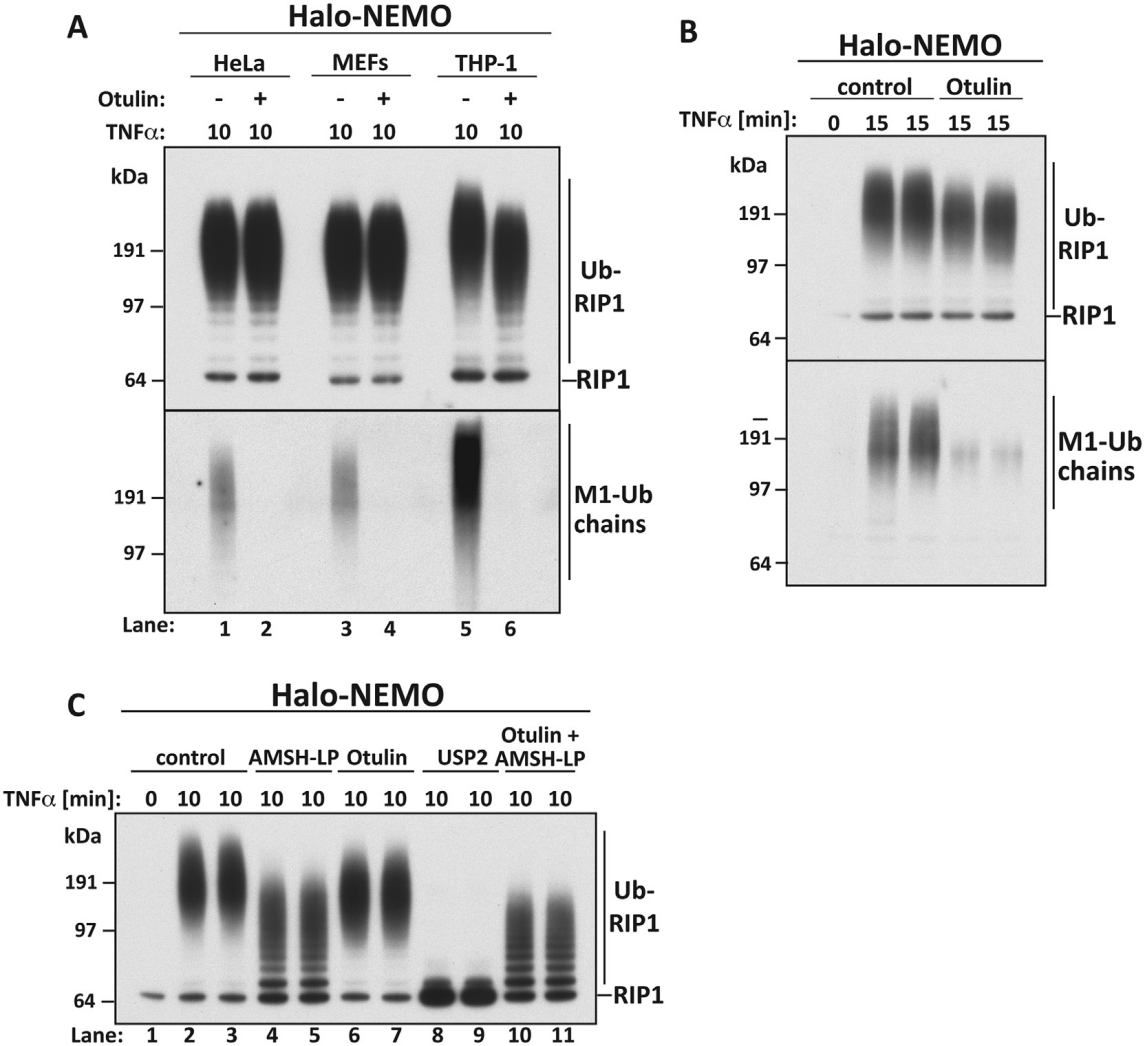
**TNFα induces the formation of hybrid ubiquitin chains containing M1-Ub linkages**. (**A**) HeLa cells, MEFs and THP1 monocytes were stimulated for 10 min with TNFα and ubiquitylated proteins captured from 2 mg cell extract protein and incubated with or without 1.0 μM Otulin as in Fig. 1. The samples were subjected to SDS/PAGE and immunoblotted with an antibody that recognizes all forms of RIP1. (**B**) As in A, except that human primary macrophages were stimulated with TNFα. (**C**) As in Fig. 1B, except that the samples were immunoblotted with the RIP1 antibody.

Following TNFα-stimulation, only a minor proportion of the RIP1 in THP1 monocytes (and other cells) undergoes ubiquitylation [22]. We found that Halo-NEMO beads captured the ubiquitylated species of RIP1 (Fig. 2A). Incubation with Otulin consistently reduced the size of the ubiquitin chains attached to RIP1 in THP1 monocytes, but without generating any mono-ubiquitylated or unmodified RIP1 (Fig. 2A, compare lanes 5 and 6). This finding demonstrated that not only were M1-Ub linkages present in the ubiquitin chains attached to RIP1, but also that they were attached covalently to another type of ubiquitin linkage(s) that was itself attached to RIP1. Similar observations were made in extracts of primary human macrophages (Fig. 2B). However, treatment with Otulin did not detectably alter the electrophoretic mobility of the ubiquitylated-RIP1 captured by Halo-NEMO beads from the extracts of TNFα-stimulated HeLa cells or immortalized MEFs (Fig. 2A, lanes 1–4). This may be due to the small number of M1-Ub linkages formed when these cells are stimulated with TNF, as compared to THP1 monocytes (Fig. 2A, bottom panel). Alternatively, or in addition, the effect of Otulin may be obscured because RIP1 molecules lacking M1-Ub linkages contain more ubiquitin linkages of other types. Consistent with this interpretation, RIP1 immunoprecipitated from the extracts of TNFα-stimulated MEFs was recently reported to contain low levels of M1-Ub together with much larger quantities of K63-Ub chains [29].

We also incubated the Halo-NEMO beads with AMSH-LP to cleave all the attached K63-Ub chains (Fig. 1B). Treatment with AMSH-LP plus Otulin generated much smaller ubiquitylated species of RIP1 than those generated by incubation with Otulin alone (Fig. 2C, compare lanes 6 and 7 with 10 and 11), indicating that some of the ubiquitin molecules attached to RIP1 were linked via Lys63 of ubiquitin. However, even after complete hydrolysis of the K63-Ub and M1-Ub linkages with Otulin plus AMSH-LP, RIP1 still migrated as a ladder of bands containing a number of ubiquitin molecules, which could be hydrolysed by the non-specific DUB USP2. These ubiquitylated species could represent multi-mono-ubiquitylated forms of RIP1 in which mono-ubiquitin is attached covalently to two, three or more lysine residues in the protein, and/or smaller ubiquitin oligomers of a distinct linkage type. Consistent with the latter possibility, the ubiquitin chains attached to the endogenous RIP1 after TNFα-stimulation have been reported to contain K11-Ub and K48-Ub linkages, as well as K63-Ub linkages [30].

### 3.3 Met1-linked ubiquitin chains are attached directly to TNFR1 in THP1 cells

**Fig. 3:**
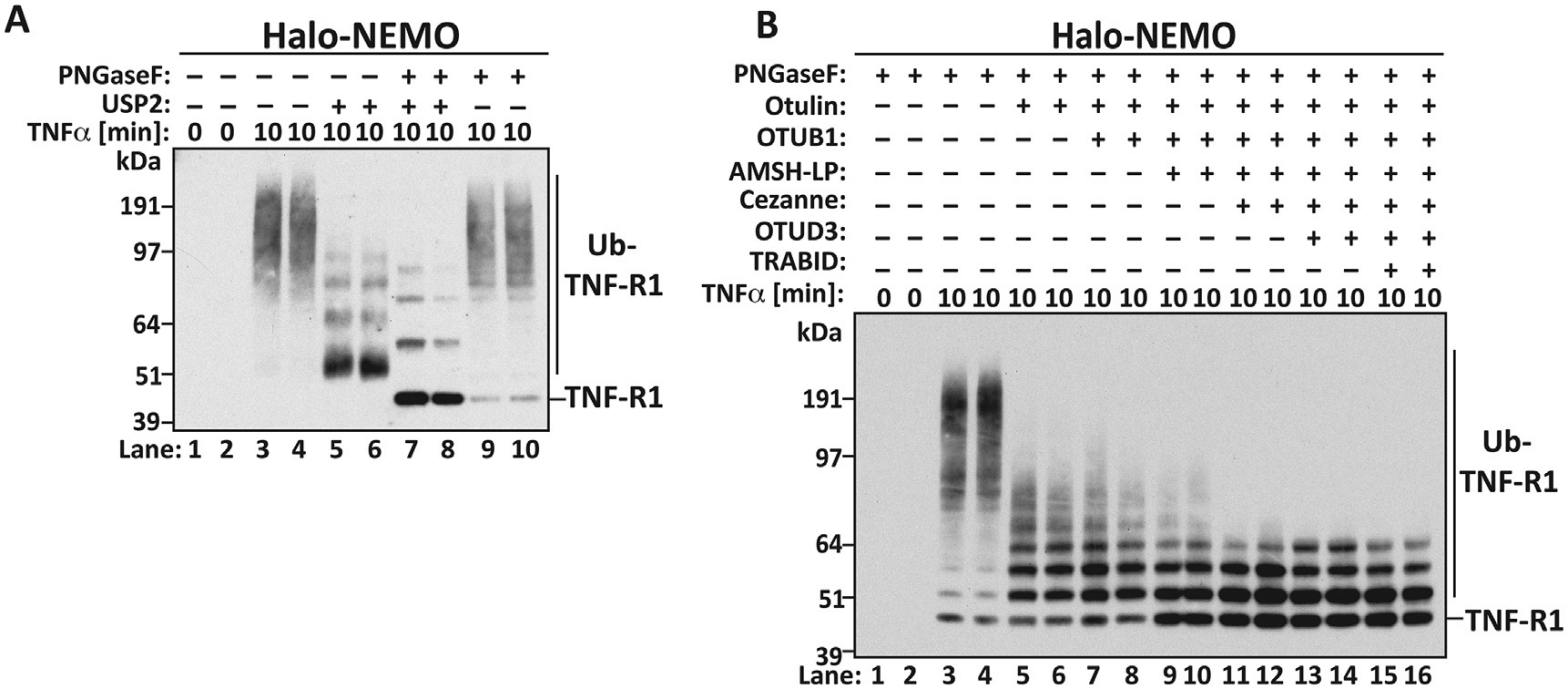
**TNFα-stimulated attachment of M1-Ub chains to TNF Receptor 1**. (**A**) THP-1 monocytes were stimulated for 10 min with 10 ng/ml TNFα and ubiquitin chains were captured from 2.5 mg of cell extract protein on Halo-NEMO beads. Samples were treated for 1 h with λ-PPase in the presence or absence of USP2 (1 μM) and/or PNGase F (500 Units). The TNFR1 protein was identified by immunoblotting. (**B**) As in A except that ubiquitylated forms of TNFR1 captured on immobilized Halo-NEMO were treated with λ-PPase (100 Units), PNGase F (500 Units) and the DUBs Otulin (1 μM), OTUB1 (2 μM), AMSH-LP (0.25 μM), Cezanne (5 μM), OTUD3 (2 μM), or TRABID (1 μM) as indicated. The samples were subjected to SDS-PAGE and immunoblotted with an antibody raised against TNFR1.

Interestingly, we found that Halo-NEMO beads captured high molecular mass forms of TNFR1 from the extracts of TNFα-stimulated THP1 monocytes, but not from the extracts of unstimulated cells (Fig. 3A, lanes 1–4). Treatment of the Halo-NEMO beads with USP2 converted these high molecular mass forms to much faster migrating species, indicating that TNFα had triggered the ubiquitylation of TNFR1. The electrophoretic mobility of TNFR1 was increased further by incubation of the Halo-NEMO beads with USP2 plus the PNGase F, which hydrolyses carbohydrate moieties attached to asparagine residues on TNFR1 [31]. PNGase F and phageλ protein phosphatase (λPPase) were therefore included in all subsequent experiments to remove covalently bound carbohydrate and phosphate from TNFR1, which might otherwise complicate interpretation of the results.

We found that treatment with Otulin largely converted TNFR1 to a discrete ladder of bands that corresponded to mono-, di- and tri-ubiquitylated species, together with smaller amounts of diffuse species of higher molecular mass (Fig. 3B). The latter were reduced by further treatment with AMSH-LP to hydrolyse K63-Ub linkages and almost completely by treatment with AMSH-LP plus Cezanne, a DUB that cleaves K11-Ub linkages relatively specifically. The further addition of OTUB1 (which hydrolyses K48-Ub linkages specifically), OTUD3 (which hydrolyses many ubiquitin linkages including K6-Ub chains) and TRABID (which hydrolyses K29-Ub and K33-Ub linkages) had little effect [23], [32]. The simplest interpretation of these results is that the mono-, di- and tri-ubiquitylated species generated by Otulin are multi-mono-ubiquitylated forms of TNFR1, implying that, in contrast to RIP1, M1-Ub chains are attached directly to the mono-ubiquitylated TNFR1 and not indirectly by attachment to another type of ubiquitin chain. In addition, lesser amounts of K11-Ub and K63-Ub oligomers also appear to be attached to the TNFR1.

### 3.4 Activation of the TRIF-dependent signaling network induces the formation of K63/M1-Ub hybrids

**Fig. 4:**
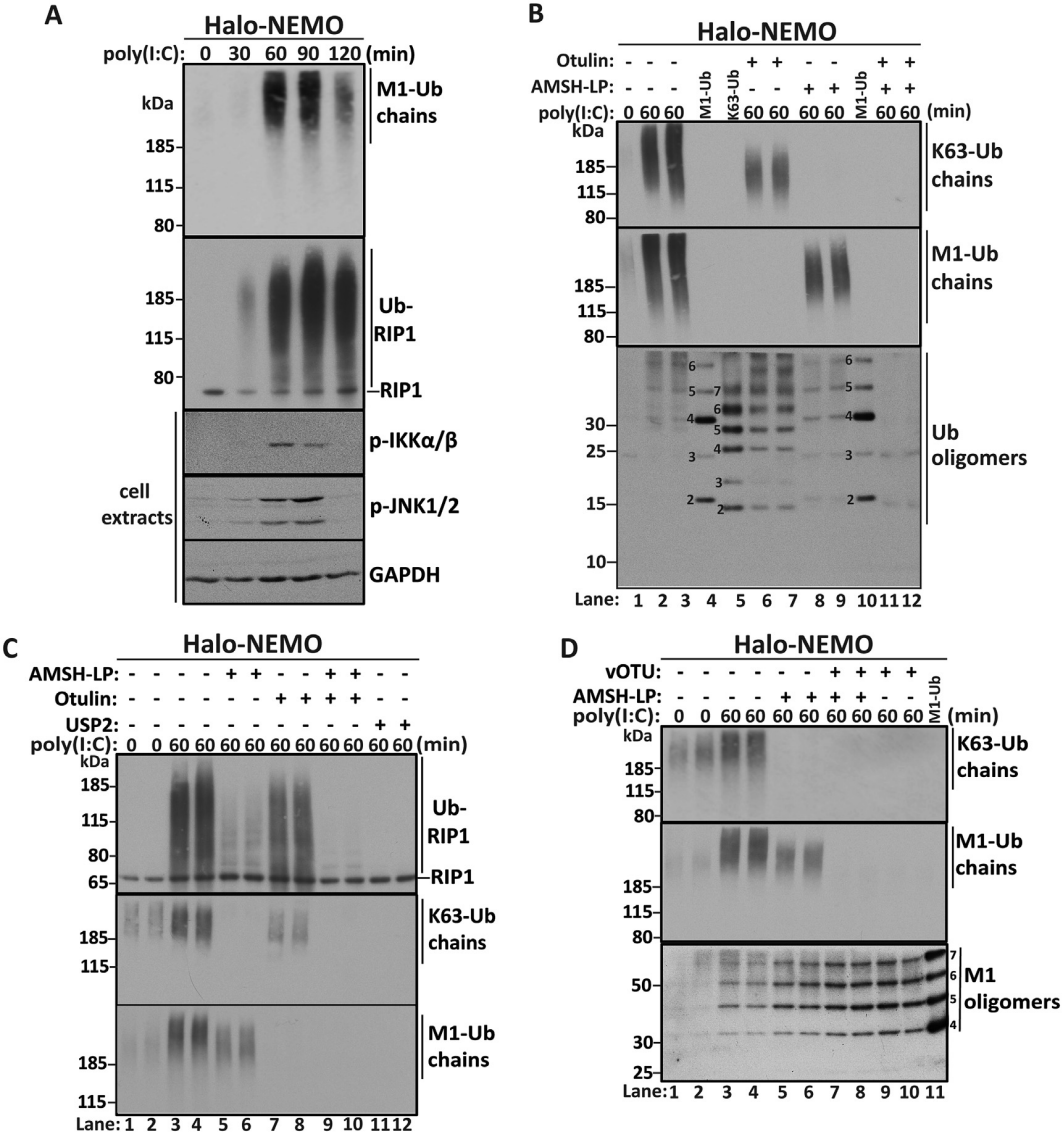
P**oly(I:C) induces K63/M1-Ub hybrid chain formation in BMDM**. (**A**) BMDM were stimulated with 10 μg/ml poly(I:C) for the times indicated and ubiquitin chains were captured from 2 mg of cell extract protein with Halo-NEMO beads. The captured M1-Ub chains and ubiquitylated RIP1 were identified by immunoblotting. Aliquots of the cell extract (20 μg protein) were subjected to SDS-PAGE and immunoblotting with antibodies that recognize the active phosphorylated forms of IKKα, IKKβ, JNK1 and JNK2, and with GAPDH as a loading control. (**B**) Similar to (A) except that, after the Halo-NEMO pull-down, samples were treated for 1 h with λ-PPase (100 units) in the absence (−) or presence (+) of Otulin (1.0 μM) and/or AMSH-LP (0.2 μM). The samples were subjected to SDS/PAGE and proteins of >65 kDa immunoblotted for K63-Ub and M1-Ub chains. Proteins <50 kDa were immunoblotted with an antibody that recognizes all forms of ubiquitin to detect the small Ub oligomers released by treatment with DUBs. M1-Ub oligomeric standard marker proteins (12 ng - lanes 4 and 10) and K63-Ub oligomeric standards (5 ng - lane 5) were included to identify the small Ub oligomers formed after treatment with AMSH-LP or Otulin. (**C**) Similar to B except that, after incubation with Otulin and/or AMSH-LP, the gels were immunoblotted with a RIP1 antibody, as well as with antibodies that recognize M1-Ub and K63-Ub chains. (**D**) Similar to B, except that the large and small M1-Ub oligomers were detected by immunoblotting before and after treatment with the deubiquitylases AMSH-LP and/or vOTU (0.1 μM).

dsRNA formed during viral replication activates TLR3, which recruits TRIF. This initiates signal transduction pathways, one of which leads to the ubiquitylation of RIP1 and activation of the canonical IKK complex. In the present study we stimulated murine BMDM with the synthetic dsRNA mimetic poly(I:C) and captured the ubiquitin chains and ubiquitylated proteins present in the cell extracts on Halo-NEMO beads. Stimulation with poly(I:C) induced the formation of M1-Ub chains, the ubiquitylation of RIP1 and the activation of the canonical IKK complex and JNK1/JNK2 that were maximal between 60 and 90 min (Fig. 4A). Poly(I:C) also induced the formation of K63-Ub chains after 60 min (Fig. 4B). Subsequent experiments were therefore carried out after 60 min stimulation.

To investigate whether poly(I:C) had stimulated the formation of K63/M1-Ub hybrids, we again studied the formation of the small ubiquitin oligomers that were generated when Halo-NEMO beads were incubated with AMSH-LP to cleave K63-Ub chains or with Otulin to cleave M1-Ub chains (Fig. 4B, top two panels). The complete hydrolysis of M1-Ub chains reduced the size and amount of the largest K63-Ub chains (Fig. 4B top panel), and induced the appearance of small Ub oligomers containing 2–7 ubiquitins that co-migrated precisely with K63-Ub oligomeric standards (Fig. 4B bottom panel, compare lanes 6 and 7 with lane 5) and which could be cleaved by AMSH-LP (Fig. 4B, lanes 11 and 12). Conversely, the complete hydrolysis of K63-Ub chains reduced the size and amount of the largest M1-Ub chains (Fig. 4B middle panel), and increased the amount of the small Ub oligomers that co-migrated with M1-Ub oligomeric standards (Fig. 4B bottom panel, compare lanes 8 and 9 with 10) and could be cleaved by Otulin (Fig. 4B, lanes 11 and 12). These experiments demonstrate that activation of the poly(I:C)/TLR3/TRIF pathway triggers the formation of K63/M1-Ub hybrids.

Importantly, the bulk of the M1-Ub chains formed in response to poly(I:C) and captured from the cell extracts on Halo-NEMO beads (>200 kDa) were larger than the ubiquitin chains attached to RIP1 (Fig. 4C, top and bottom panel), implying that most of the M1-Ub chains formed in response to poly(I:C) were not attached covalently to RIP1. Consistent with this inference, most of the ubiquitin chains attached to RIP1 disappeared upon incubation with AMSH-LP, while treatment with Otulin had relatively little effect (Fig. 4C, top panel). These observations suggest that most of the ubiquitin chains attached to RIP1 are linked via Lys63, although a small number of M1-Ub linkages may also be present.

Interestingly, the high molecular mass M1-Ub chains still remaining after incubation with AMSH-LP disappeared when the Halo-NEMO beads were incubated with AMSH-LP plus vOTU, or with vOTU alone (Fig. 4D, middle panel). vOTU is a virally-encoded DUB, which hydrolyses all Ub-linkage types, except for M1-Ub and K29-Ub linkages [23]. Treatment with AMSH plus vOTU increased the formation of the small M1-Ub oligomers compared to AMSH-LP alone (Fig. 4D, bottom panel), suggesting that the large M1-Ub chains still present after treatment with AMSH-LP may comprise hybrid ubiquitin chains containing M1-Ub and another Ub linkage(s) type distinct from K63-Ub chains. However, it cannot be excluded that these M1-Ub oligomers were attached directly to a mono-ubiquitylated protein(s), since vOTU also cleaves the first ubiquitin moiety attached to proteins.

### 3.5 Activation of NOD1 signaling induces the formation of K63/M1-Ub hybrids

**Fig. 5:**
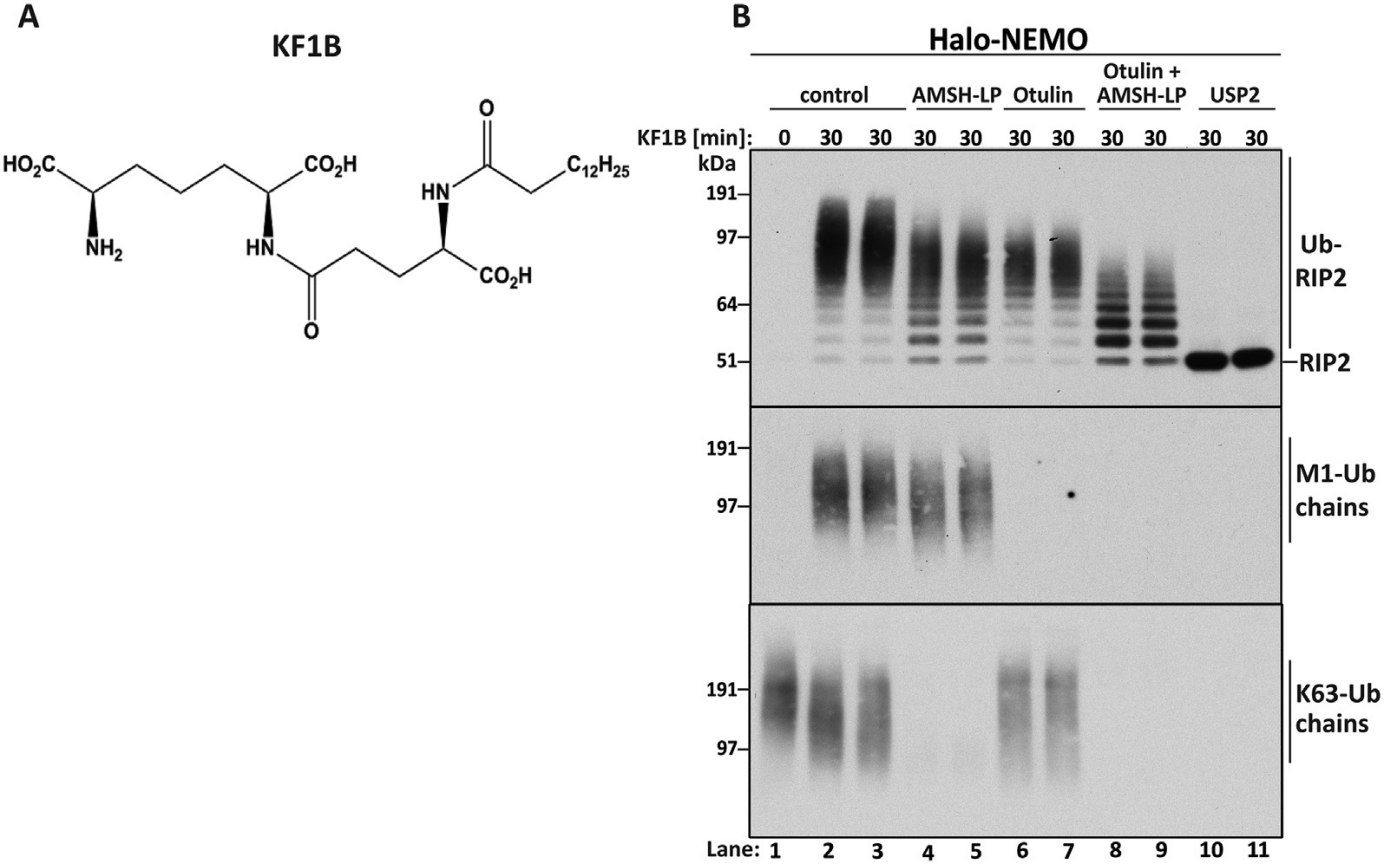
**KF1B induces the NOD1-dependent formation of hybrid ubiquitin chains attached to RIP2 that contain K63 and M1-Ub linkages**. (**A**) The structure of KF1B. (**B**) RAW macrophages were stimulated for 30 min with 25 μM KF1B and ubiquitin chains captured from 2 mg of cell extract protein with SDS-washed Halo-NEMO beads (see Methods). The samples were treated for 1 h with λPPase (100 units) plus AMSH-LP (0.05 μM) and/or Otulin, or USP2 (1.0 μM). The samples were subjected to SDS/PAGE and immunoblotted with antibodies that recognize RIP2, M1-Ub chains or K63-Ub chains.

NOD1 and NOD2 are cytosolic receptors that sense the presence of intracellular bacteria. Upon activation by peptides derived from bacterial peptidoglycans, the NOD1/2 receptors interact with RIP2 and XIAP, another member of the IAP family of E3 ubiquitin ligases that is encoded by an X chromosome-linked gene. This leads to the ubiquitylation of RIP2 [33], and the activation of TAK1 [34] and NF-κB [35]. Here, we stimulated the RAW264.7 macrophage-like cell line with the NOD1 agonist KF1B (Fig. 5A) and captured the ubiquitylated forms of RIP2, M1-Ub chains and associated K63-Ub chains from the cell extracts on Halo-NEMO beads (Fig. 5B). Incubation with Otulin decreased the size of the ubiquitin chains attached to RIP2, but without causing any conversion to mono-ubiquitylated or deubiquitylated species. (Fig. 5B, compare lanes 2–7). These experiments show that the ubiquitylated RIP2 contains M1-Ub linkages that are not attached directly to a lysine residue on RIP2, but are attached to RIP2 indirectly via another type of ubiquitin linkage(s). Similar observations have been made previously in RAW macrophages after stimulation with the NOD2 activator muramyl dipeptide [17]. Incubation of the Halo-NEMO beads with Otulin plus AMSH-LP largely converted RIP2 to a mixture of mono-ubiquitylated, diubiquitylated and tri-ubiquitylated species (Fig. 5B, lanes 8 and 9) that may be multi-monoubiquitylated forms of RIP2, or RIP2 containing a further type of Ub linkage. Taken together, these experiments indicate that hybrid ubiquitin chains containing both M1-Ub and K63-Ub linkages are attached to RIP2 when the NOD1 receptor is activated.

### 3.6 The role of Met1-linked ubiquitin chains in activating the canonical IKK complex in different innate immune signaling pathways

**Fig. 6:**
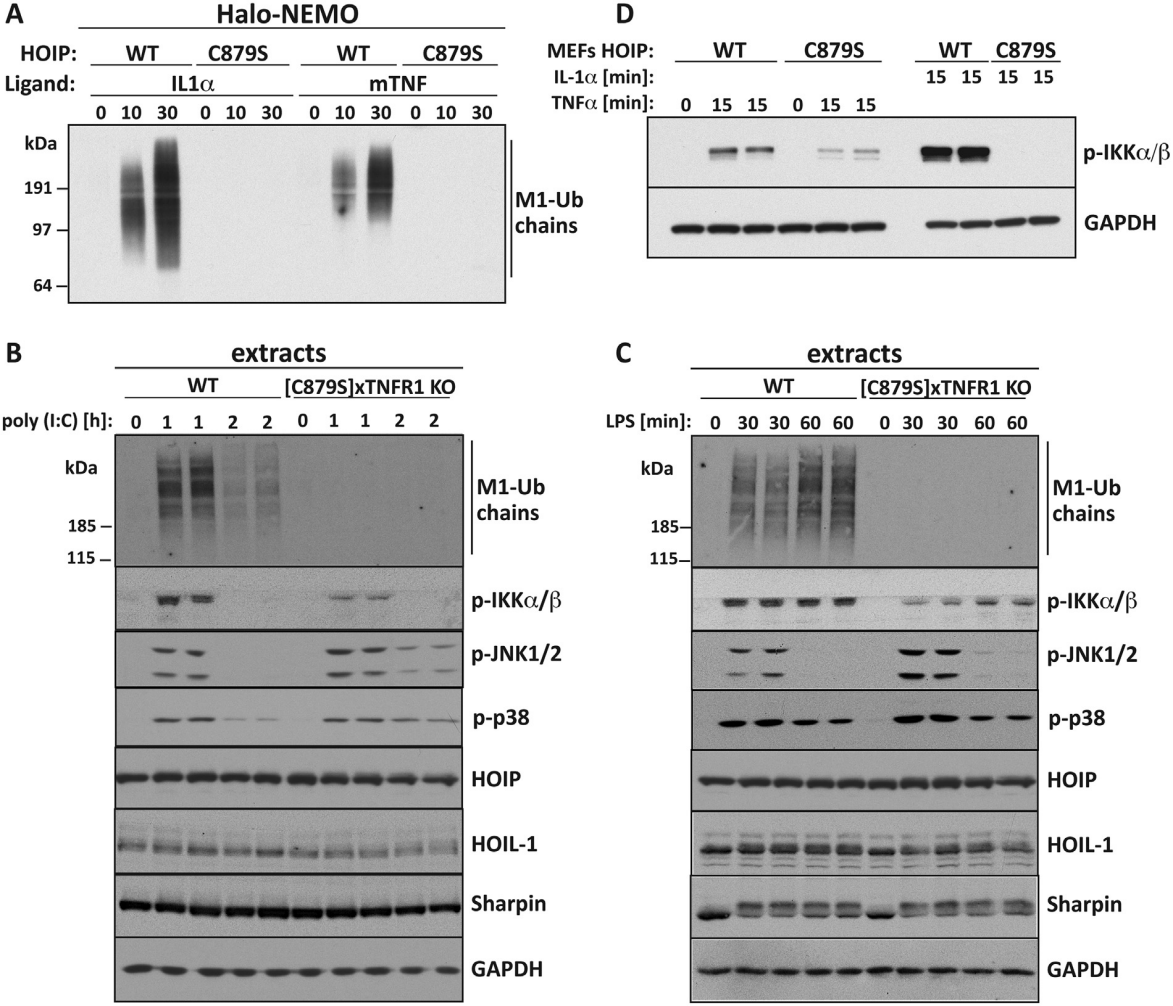
**M1-Ub chain formation and activation of the canonical IKK complex in cells from knock-in mice expressing an E3 ligase-inactive mutant of HOIP**. (**A**) MEFs expressing either wild-type (WT) HOIP or the E3 ligase-inactive HOIP[C879S] mutant ([C879S]) were stimulated with either 5 ng/ml IL-1α or 10 ng/ml mTNFα for the times indicated. M1-Ub chains captured from the cell extracts with Halo-NEMO were identified by immunoblotting with a specific antibody. (**B, C**) Macrophages derived from the foetal livers of WT or HOIP[C879S] knock-in mice were stimulated with poly(I:C) (B) or LPS (C) for the times indicated. Cell extract (20 μg protein) was subjected to SDS-PAGE and immunoblotted with antibodies that recognize M1-Ub chains (top panel), the activated forms of IKKα, IKKβ, JNK1 and JNK2, the three components of LUBAC (HOIP, HOIL-1 and Sharpin) and GAPDH as a loading control. (**D**) As in A except that the extracts (20 μg protein) were immunoblotted with antibodies that recognize the active phosphorylated form of IKKα and IKKβ and for GAPDH as a loading control.

HOIP is the catalytic component of LUBAC. We have generated MEFs from embryos that express the catalytically inactive HOIP[C879S] mutant instead of wild type HOIP, and showed that they do not form any M1-Ub chains in response to IL-1α [9]. Here, we show that MEFs from the HOIP[C879S] embryos also failed to produce M1-Ub chains upon stimulation with TNFα (Fig. 6A). Similarly, macrophages derived from the foetal livers of these mice did not produce any M1-Ub chains after stimulation with poly(I:C) (Fig. 6B) or LPS (Fig. 6C). These results demonstrate that HOIP, is the only E3 ligase generating M1-Ub chains when the IL-1R, TNFR1, TLR3 and TLR4 receptors are activated.

We have also shown that the IL-1α-dependent activation of IKKα and IKKβ is greatly reduced in MEFs from HOIP[C879S] embryos, as shown by the drastic decrease in the IL-1α-dependent phosphorylation of the activation loops of these protein kinases ([9] and see also Fig. 6D). In contrast, the TNFα-dependent activation of the IKKs was only reduced by about 50% in MEFs from the HOIP[C879S] embryos (Fig. 6D). The poly(I:C)-stimulated (Fig. 6B) or LPS-stimulated (Fig. 6C) phosphorylation of the canonical IKK complex were also reduced by about 50% in macrophages from the HOIP[C879S] embryos.

## 4 Discussion

In this paper, we show that hybrid ubiquitin chains containing M1-Ub and K63-Ub linkages are produced rapidly when the TNFR1-TRADD, TLR3-TRIF and NOD1-RIP2 signaling pathways are activated. In conjunction with our earlier studies on the IL-1R/TLR-MyD88 signaling network [9] and other studies on NOD2 signaling [17], these observations demonstrate that the rapid formation of hybrid ubiquitin chains containing M1-Ub linkages is a general feature of innate immune signaling systems. All these networks trigger the activation of the TAK1 and canonical IKK complexes, so that one role of these hybrid chains may be to co-recruit these kinase complexes and so facilitate the TAK1-dependent activation of the IKKs, as mentioned in the Introduction.

However, the K63/M1-hybrids are likely to serve several other functions. For example, A20-binding inhibitor of NF-κB 1 (ABIN1), which possesses a similar ubiquitin-binding domain to NEMO, restricts the activation of TAK1 and the canonical IKK complex by binding to the K63/M1-Ub hybrids. As a consequence, TAK1 and IKK become hyper-activated, and pro-inflammatory cytokines are overproduced, when the TLR-MyD88 network and NOD2 signaling network are activated in myeloid cells from knock-in mice expressing the ubiquitin-binding-defective ABIN1[D485N] mutant. These mice develop a disease that closely resembles Type III and Type IV lupus in humans, termed lupus nephritis, and this can be prevented by crossing the ABIN1[D485N] mice to MyD88 knock-out mice [36]. Moreover, the ABIN1-interacting protein A20, which also restricts the activation of TAK1 and the canonical IKK complex, contains seven zinc fingers at its C-terminus, two of which are reported to bind specifically to K63-Ub linkages and M1-Ub linkages, respectively [37], [38], [39], [40]. Thus ternary complexes formed between ABIN1, A20 and K63/M1-Ub hybrids have critical roles in preventing the overproduction of inflammatory mediators. It has also been reported that the covalent attachment of M1-Ub oligomers to K63-Ub oligomers suppresses the rate at which K63-Ub oligomers are hydrolysed in vitro by the deubiquitylase activity of A20, which is located near the N-terminus of the protein [29]. Thus hybrid ubiquitin chains may affect the rates at which K63-Ub and M1-Ub chains are deubiquitylated and so control the duration of the innate immune response.

The TNFα-stimulated activation of the canonical IKK complex was only reduced by about 50% in MEFs from HOIP[C879S] embryos that cannot produce any M1-Ub linkages. This is similar to observations made previously in human and mouse fibroblasts lacking expression of one or more components of LUBAC [41], [42]. A similar reduction in IKK activation was observed when liver macrophages from HOIP[C879S] mice were stimulated with poly(I:C) (Fig. 6B) or LPS (Fig. 6C) to activate the TLR3-TRIF and TLR4-TRIF/MyD88 signaling network. This contrasts with the more drastic reduction in IKK activation observed when MEFs from HOIP[C879S] embryos were stimulated with IL-1α ([9] and see also Fig. 6D). The reason why the formation of M1-Ub chains appears to be more critical for IL-1α-dependent IKK activation than TNFR1-dependent or TLR3-dependent IKK activation is unclear. It might be explained by differences in the topology of the K63/M1-Ub hybrids that are formed in response to the different stimuli and/or by the presence of a further type of ubiquitin linkage in the K63/M1-Ub-containing hybrid molecules that are formed in response to TNFα or poly(I:C).

The only protein detected in this study in which the M1-Ub chains did not seem to be attached covalently to another type of ubiquitin linkage was TNFR1, most of the M1-Ub chains apparently being linked directly to the first ubiquitin moiety attached to TNFR1. While our studies were in progress, two other laboratories also reported that M1-Ub chains were attached covalently to the TNFR1 [29], [43], although the mechanism of attachment was not investigated. Since MEFs from HOIP-deficient embryos are hypersensitive to TNFα-induced cell death, these investigators suggested that the ubiquitylation of TNFR1 may function mainly to regulate cell death. In contrast, the TNFα-stimulated formation of hybrid ubiquitin chains containing M1-Ub, K63-Ub and other Ub-linkage types that are attached to RIP1 may function to activate the TAK1 and canonical IKK complexes, which inhibits TNFα-induced apoptosis by activating NF-κB.

## Funding

This work was supported by the Wellcome Trust [grant WT100294] and by AstraZeneca, Boehringer Ingelheim, GlaxoSmithKline, Janssen Pharmaceutica and Merck-Serono.
